# Supplementary material for: Skeletal Muscle Dysfunction in Experimental Pulmonary Hypertension
Source: Int J Mol Sci. 2022 Sep 18;23(18):10912. doi: 10.3390/ijms231810912 (PMC9501428; doi:10.3390/ijms231810912)
Supplement: Supplementary file 1 [file ijms-23-10912-s001.zip › ijms-1889225-supplementary.pdf]

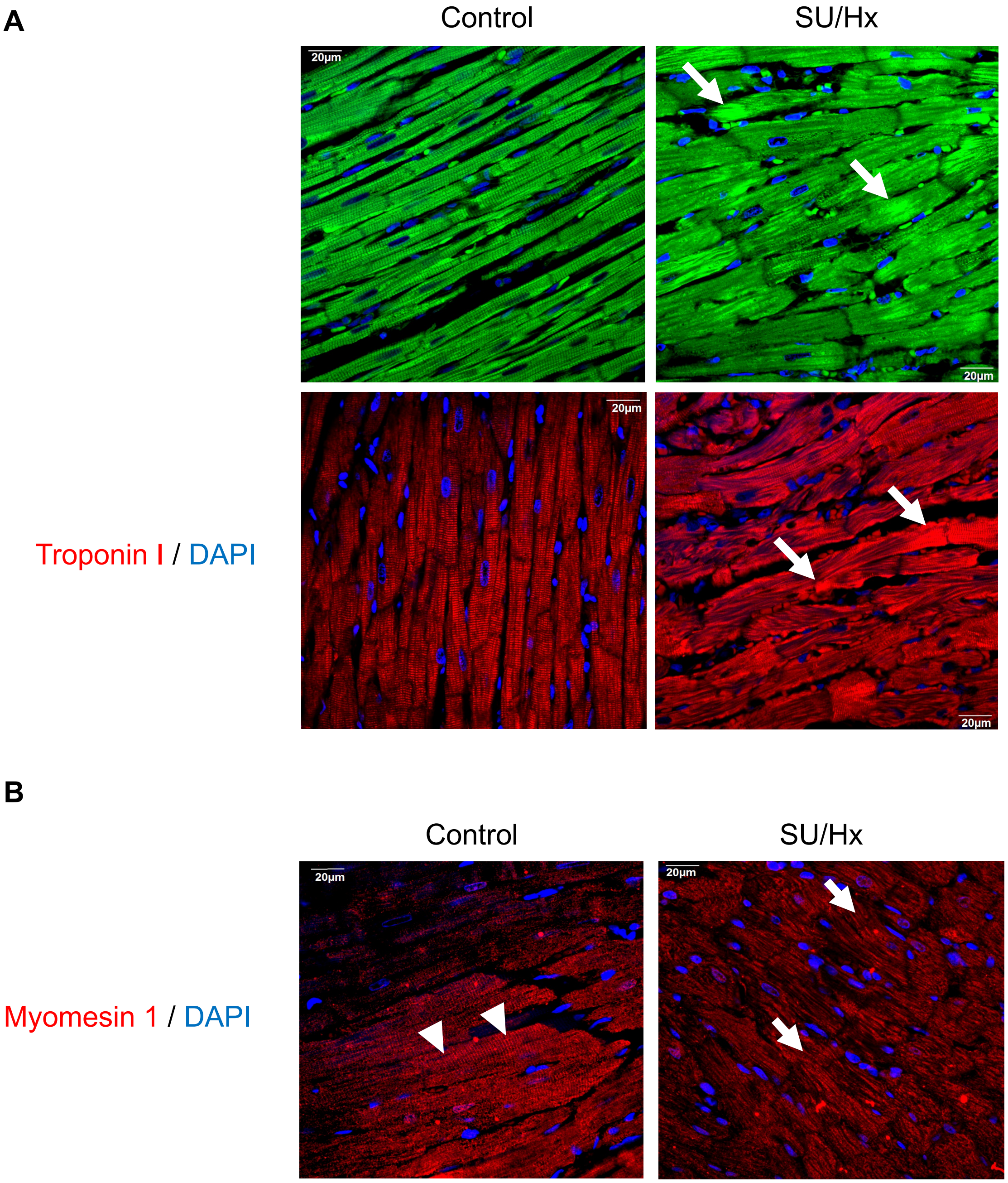

Supplementary figure S1.

Supplementary figure S1. (A) Disarray of sarcomeric organization in SU/Hx-induced PH rats. Paraffin- embedded sections of whole heart specimens stained with desmin (Z-disc) and cardiac troponin-I specific antibodies showing a compromised and disorganized sarcomeric organization in SU/Hx-induced PH rats compared to controls. Staining for desmin and cardiac troponin-I (respectively) revealed chaotic organization with fewer striations with aggregate-like structures (arrows) in SU/Hx-induced PH rats when compared to controls. **(B)** Disarray of sarcomeric M-lines in SU/Hx-induced PH rats. Paraffin- embedded sections of whole heart specimens stained with myomesin 1 specific antibody showing a compromised and disorganized sarcomeric organization in SU/Hx-induced PH rats compared to controls. Staining for myomesin 1 revealed chaotic organization with fewer striations (arrows) in SU/Hx-induced PH rats when compared to well-formed M-lines in controls (arrowheads). Scale bar, 20 µm. Nuclei were visualized with DAPI staining.

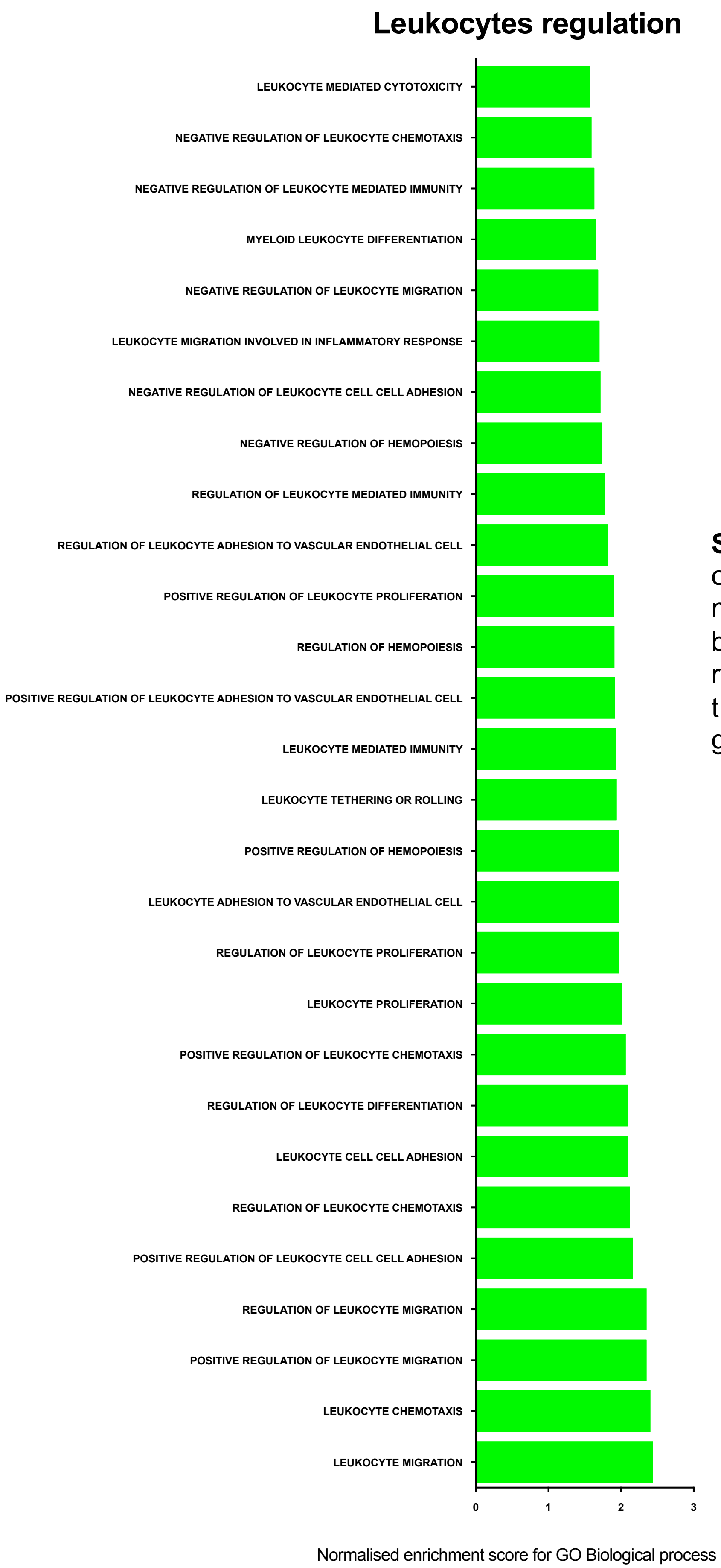

Supplementary figure S2. Gene ontology enrichment analysis of the most significantly upregulated biological processes leukocytes regulation related terms in diaphragm transcripts between SuHx and control groups by false discovery rate (FDR).

Supplementary figure S2.

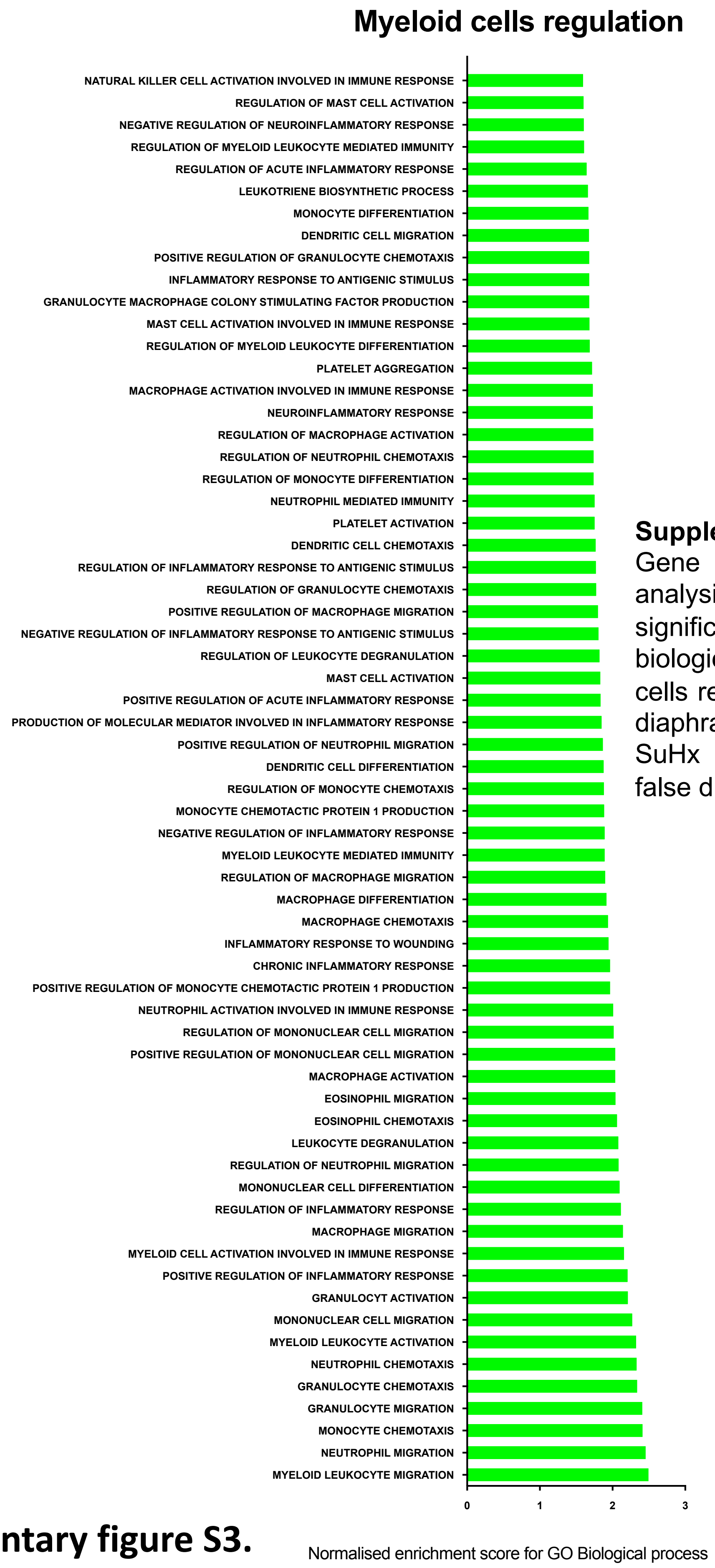

Supplementary figure S3. Gene ontology enrichment analysis of the most significantly upregulated biological processes myeloid cells regulation related terms in diaphragm transcripts between SuHx and control groups by false discovery rate (FDR).

Supplementary figure S3.

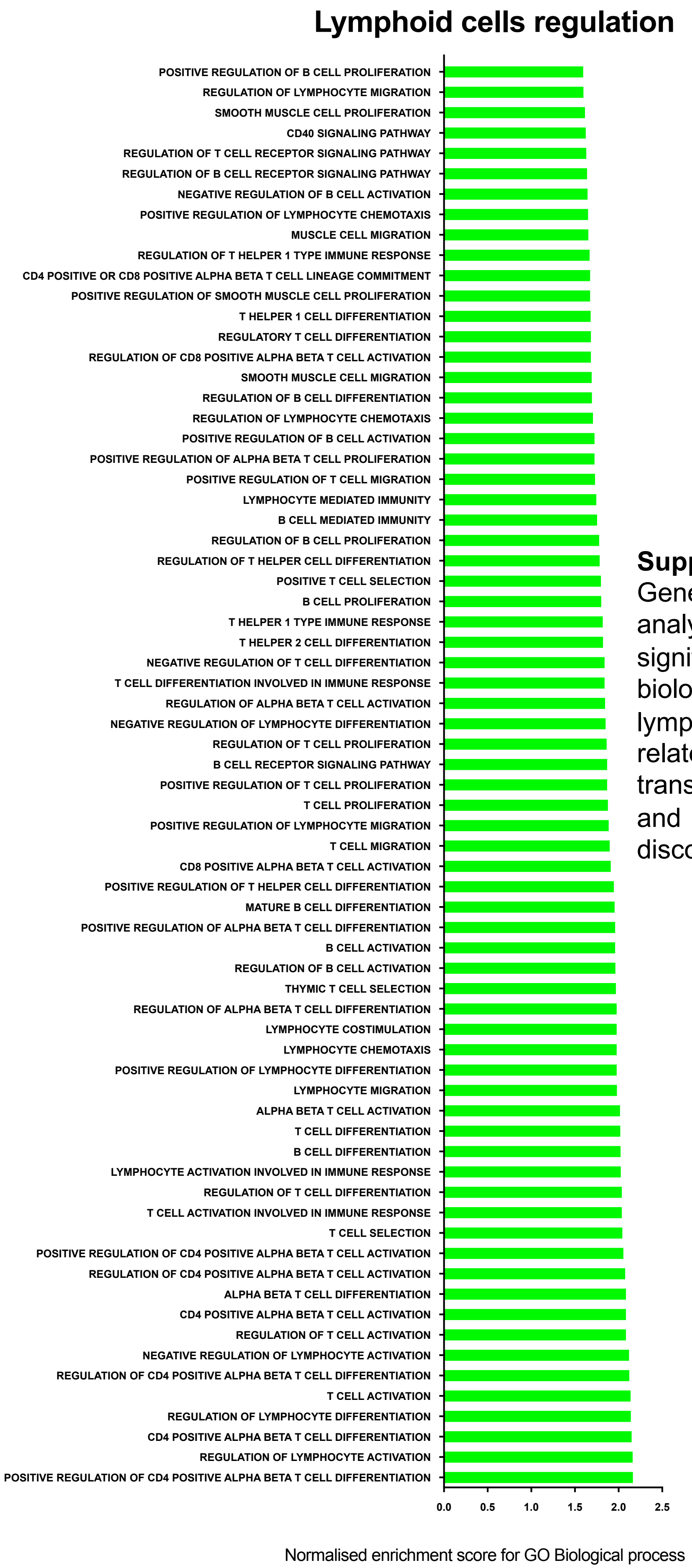

Supplementary figure S4. Gene ontology enrichment analysis of the most significantly upregulated biological processes lymphoid cells regulation related terms in diaphragm transcripts between SuHx and control groups by false discovery rate (FDR).

Supplementary figure S4.

Gene	Accession No.	Forward primer (5'-3')	Reverse primer (5'-3')
Actn2	NM_001170325.1	ACCCAGGAGCAGATGAAT	CAGCTTCACCCAAGTCATAG
Cyp1a1	NM_012540.3	TAACTCTTCCCTGGATGCCTTCAA	GTCCCGGATGTGGCCCTTCTCAAA
Cyp1b1	NM_000104.4	GCTCAGCCACAACGAGGAGTTC	CTGGTAAAGAAGATGAGCAGC
Fbxo32 (Atrogin-1)	NM_133521.1	TAAGGAGCGCCATGGATA	CAGCTCCAACAGCCTTAC
FoxO1	NM_001191846.3	GATAAGGGCGACAGCAACAG	GATTGAGCATCCACCAAGAAC
GAPDH	NM_017008.4	GACATGCCGCCTGGAGAAAC	AGCCCAGGATGCCCTTTAGT
Glut1	NM_138827.1	TTCGGCTTAGACTCCATCA	GAAGGGCAACAGGATACAC
Glut4	NM_012751.1	CTCAATGGTTGGGAAGGAAA	CCGTCCGAGAATGAGTATCT
Myh1	XM_008767787.1	GGAAGTAAAGGCCAAGAGTG	CTTGGCTTCCTGTTCTTCTT
Myh4	NM_019325.1	CTCCCGCTTCGGTAAATTC	GGTGACTCTGGACTTCTCTA
Myogenin	NM_017115.3	GTACCCAGTGAATGCAACTC	CTGCGAGCAAATGATCTCC
Nup133	XM_008772670.2	GTCTTCGATACACAAGGAGAC	CTTCTGCTAACAGGGACATAC
PGC-1a (Ppargc1a)	NM_031347.1	GGGATGATGGAGACAGCTA	TTCATTCGACCTGCGTAAAG
Sirt1	XM_008774950.1	TAATCAGGTAGTTCCTCGGT	GGCTCTATGAAACTGTTCTGG
Tfam	NM_031326.1	CCTGTCAGCCTTATCTGTATTC	TTTGGGTAGCTGTTCTGTAG
Tnni1	NM_017184.1	CACAACACGAGAGAGATCAAG	TTGTGTTTGGAACCCAGTAG
Trim63 (Murf-1)	NM_133521.1	TAAGGAGCGCCATGGATA	CAGCTCCAACAGCCTTAC

Supplementary Table S1.

Supplementary Table S1. Primers for rat genes in quantitative real-time PCR.
